# Identifying single-item faked responses in personality tests: A new TF-IDF-based method

**DOI:** 10.1371/journal.pone.0272970

**Published:** 2022-08-25

**Authors:** Alberto Purpura, Dora Giorgianni, Graziella Orrù, Giulia Melis, Giuseppe Sartori

**Affiliations:** 1 IBM Research Europe, Dublin, Ireland; 2 Department of General Psychology, University of Padua, Padua, Italy; 3 Department of Surgical, Medical, Molecular and Critical Area Pathology, University of Pisa, Pisa, Italy; Sapienza, University of Rome, ITALY

## Abstract

Faking in a psychological test is often observed whenever an examinee may gain an advantage from it. Although techniques are available to identify a faker, they cannot identify the specific questions distorted by faking. This work evaluates the effectiveness of term frequency-inverse document frequency (TF-IDF)—an information retrieval mathematical tool used in search engines and language representations—in identifying single-item faked responses. We validated the technique on three datasets containing responses to the 10-item Big Five questionnaire (total of 694 participants, respectively 221, 243, and 230) in three faking situations. Each participant responded twice, once faking to achieve an objective in one of three contexts (one to obtain child custody and two to land a job) and once honestly. The proposed TF-IDF model has proven very effective in separating honest from dishonest responses—with the honest ones having low TF-IDF values and the dishonest ones having higher values—and in identifying which of the 10 responses to the questionnaire were distorted in the dishonest condition. We also provide examples of the technique in a single-case evaluation.

## Introduction

In this study, we address the problem of interpreting the data collected through questionnaires when responses are distorted by faking. Examinees can easily fake responses to direct questions when they find a direct advantage in doing so, and distortions may take one of two forms: faking bad and faking good. In faking bad, the examinee is likely to exaggerate or make up a psychological disorder (e.g., in criminal cases and in insurance claims; [1]. In contrast, faking good usually occurs in a setting where the respondent is expected to give highly desirable responses.

In psychological questionnaires, faking is usually controlled via the so-called control scales (e.g., the L, F, and K scales of the Minnesota Multiphasic Personality Inventory-2 (MMPI-2) [2] and the X, Y, and Z scales of the Millon Clinical Multiaxial Inventory-III; (MCMI-III) [3]. These scales detect the respondent’s propensity to depict a socially desirable profile or report hyperbolic disorders. Abnormal scores in these control scales indicate an overall tendency of the subjects to modulate their responses in the direction of social desirability (faking good) or hyperbolic psychopathology (faking bad). In general, faking is a continuous variable, and the stake and the respondent’s conscious or unconscious strategy modulate the level of faking.

For this reason, efforts have been made to develop specific tests able to flag the respondent as a faker. Although such procedures may reveal the faker with sufficient accuracy, to the best of our knowledge, no procedure has been proposed to specifically identify faked replies or reconstruct the honest response profile once a faker has been identified and only the faked profile is available. In short, a nondepressed individual who wants to appear depressed may be spotted as a faker; however, no specific procedure is available to identify faked responses.

This study aims to develop an efficient technique for spotting faked responses to individual items and not only a faking response style. We will analyze the potential of a standard information-representation model developed within the information retrieval field (i.e., term frequency-inverse document frequency (TF-IDF) [4], to identify the faked items in a psychological test. We validated the proposed model using the 10-item Big Five Test [5], a widely used short version of the Big Five personality test suited to quickly collect a psychological profile over the web. We administered this questionnaire to a group of volunteers instructed to answer twice: the first time dishonestly according to specific instructions and the second time honestly. As faking changes according to the respondent’s goal, we provided the following three faking contexts: (a) a child custody case (CC), (b) a job interview for a sales manager position (JIS), and (c) a job interview for a position in a humanitarian organization (JIHO). The faking instructions, as used here, should be considered only a gross approximation of a real high-stakes faking strategy.

In both classical test theory and item response theory (IRT), an item’s worth is evaluated with respect to the performance on that item of a sample of participants. In classical test theory, a participant’s overall responding style is not modeled–an example of a responding style is the tendency to use the full range of response alternatives or just the extreme values.

Recently, a variant of IRT has been proposed, the IRT tree model [6]. The IRT tree model differs from standard IRT, adding a further cognitive component representing the response style. Such response style is intended to represent the propensity of the responder to use extreme values in a Likert scale. Such cognitive component could be modified in order to represent the propensity of the responder to faking as proposed by [7]. The basic idea behind using an IRT tree model for faking detection in [7] is to fit a probabilistic model to represent the decision process of a respondent to a questionnaire. The model is then employed to compare honest and faked respondents’ styles and to distinguish between them.

As opposed to IRT-tree, the TF-IDF approach offers a purely data-driven and model-free solution for the task of faking-detection that only requires the collection of honest responses. This aspect, in particular, provides some important advantages for a widespread application of the proposed approach compared to IRT. In fact, a TF-IDF based approach does not require a domain expert to design the experiments and can be used following the same process regardless of the domain or type of questionnaire. Additionally, a TF-IDF based approach allows to compare the respondents’ style across different questionnaire items instead of being limited to each single item.

Our TF-IDF approach is characterized by combining a response’s relative positioning with respect to the validation sample as well as a specific subject’s response style [8]. From this perspective, it also differs from the methods that detect faking by analyzing only the extreme responses.

The original contributions of this work are:

- the proposal of a novel approach to precisely identify faking patterns in personality tests;
- a thorough evaluation of the proposed approach based on TF-IDF, with a comparison to similar techniques based on the distribution of raw response values; and
- the creation of three datasets for the development of new item-level faking-detection approaches.

## Materials and methods

TF-IDF is a standard term-weighting method used in information retrieval to compute a numeric representation of textual data based on the distribution of the terms occurring in it. It is one of the mathematical tools search engines use to retrieve web pages relevant to a user query and rank them according to their relevance. It is also used as a text representation strategy in natural language processing [8].

The TF-IDF representation strategy is based on the intuition that a certain term in a document, *d*, is representative of its general meaning proportional to (a) its term frequency (TF) within *d* and (b) its inverse frequency in the collection (IDF; i.e., a term that frequently appears in *d* and at the same time is only rarely used in others) is more representative for the content of *d* than different ones that are either not frequently used in *d* or are very frequently used also in other documents.

Formally, given a collection, *C*, of documents, the TF-IDF value for each term, *t*, in a document *d* ∈ *C* is calculated as

TF−IDF(t,d,C)=TF(d,t)*IDF(t,C), (1)

where TF (*d*, *t*) indicates the number of times a target term, *t*, appears in a document, *d*. IDF = log(*N/n*), where *N* indicates the number of documents in *C* and *n* is the number of documents in which *t* is used.

The application of TF-IDF is not only limited to the field of computer science but has also been used, for example, in cognitive neuropsychology to model semantic memory and its disorders [9] as well as brain responses to relevance in picture recognition [10].

When applied to item analysis, we compute each response’s TF-IDF in a questionnaire, *q*, considering as TF the number of times the subject provides the current response. For example, if a subject responded with a rating of 5 only to one item out of the 10 items, then TF (5, q) = 1. Indeed, for each item, the subjects could choose their degree of agreement on a 5-point Likert scale.

The IDF is computed as log(*N/n*), where *N* is the number of participants who responded to the test (for instance, 221 in the first experiment) and *n* is the number of times the participant’s response was given also by other participants to the same item. The final score associated with each test item is the product of the TF and IDF values. This adaptation of TF-IDF has high values for responses to single items that are highly atypical. From this perspective, TF-IDF behaves as an anomaly detector, indexing the very atypical responses at the single-item level (not at the subject level).

The following example illustrates how the TF-IDF could be used to spot a faker. A 10-item personality test is administered to 1,000 participants, who can express their degree of agreement on a 5-point Likert scale. Subject 1 replies “5” to Item 3 and, in total, to seven items out of 10. Regarding the whole sample’s replies to Item 3, 300 subjects reply “2”, 650 reply “3”, and 50 reply “5”. The TF-IDF score associated with Item 3 for Subject 1 is therefore computed as 7 * log (1000/50) = 9.11. This value will be high for people that provide the same—atypical—response many times in a questionnaire.

From this perspective, dishonest responses are expected to have an abnormally high TF-IDF, compared to the TF-IDF distribution of honest respondents.

**Fig 1:**
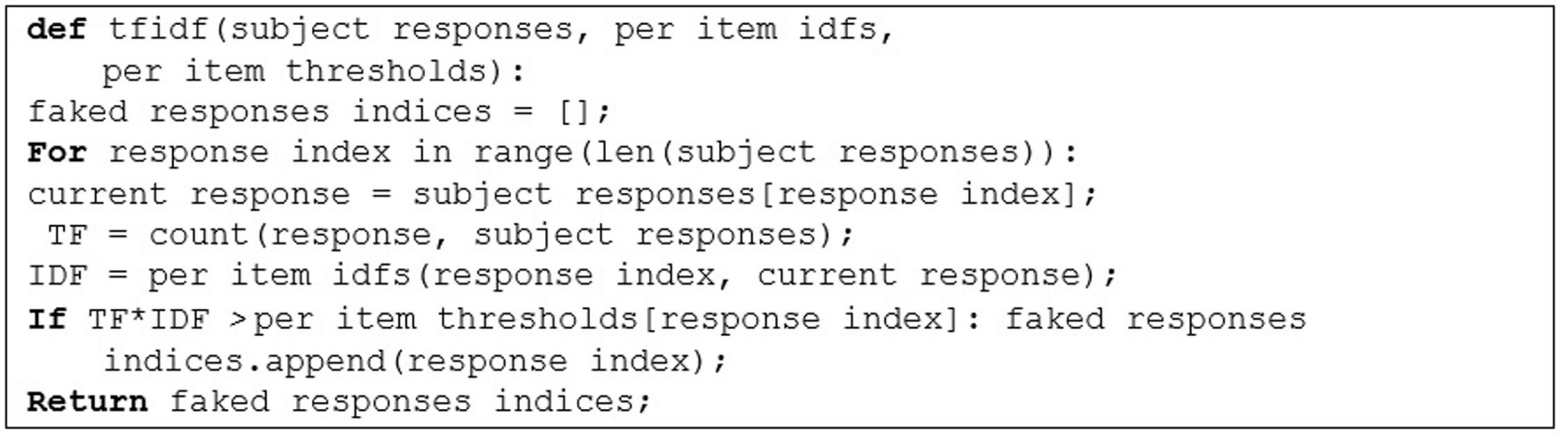
Code Snippet 1. TF-IDF for single-item faking-detection.

Fig 1 summarizes the proposed approach based on TF-IDF in the pseudo-code.

For each response a subject gives on a questionnaire, we compute its corresponding TF and IDF scores as described above. Then, if their product is higher than a certain threshold, we flag the current response as faked.

Given a training set, said threshold is selected as the percentile score in the TF-IDF values distribution (for each item) that maximizes the precision metric for faking detection on a separate validation set. It is not calculated on the raw TF-IDF scores but on the corresponding percentile of the distribution of the TF-IDF scores on a certain item. We then evaluated the threshold value following a K-fold cross-validation procedure (with K = 10). This process allowed for an evaluation of the proposed approach’s performance in terms of accuracy, precision, recall, and F1 score.

In our study, we first calculated the IDF scores associated with each item in the honest responses. Afterward, we considered a small subset of dishonest responses—as a validation set—and fine-tuned the faking detection threshold for each item based on the distribution of these scores. For example, should we observe that the value associated with the 75th percentile in the distribution of TF-IDF values in our validation set were a reliable threshold to distinguish between honest and dishonest responses for the questionnaire items, we would use said value to detect fakers for each item. We identified the 75th percentile empirically based on our data distribution. More specifically, we first computed the distribution of TF-IDF scores associated with each questionnaire item, then, we evaluated values at different percentiles of each of these distributions as potential threshold candidates to distinguish dishonest from honest responses. Finally, we selected the percentile value that maximises the F1 Score performance metric for the detection of faked response items.

When a new questionnaire item is to be evaluated for dishonesty, we rely on the previously computed set of IDF scores for the respective honest responses and on the TF scores computed for the current responses to assign a TF-IDF score to each response. Then, if the obtained TF-IDF value is higher than a certain threshold—which we validated beforehand as explained above—we report the item as faked. In other words, we rely on the TF-IDF score associated with a subject’s responses to a questionnaire as an indicator of their attitude and as a proxy to detect faking behaviors. For this purpose, TF-IDF, as proposed here, acts as an anomaly detector, identifying suspicious differences between the to-be-evaluated case and the distribution of honest respondents’ answers. Furthermore, considering various faking contexts helps us exclude potential TF-IDF approach biases to specific data distributions or situation-specific behaviors [11, 12].

To validate the technique, we conducted three experiments in which we asked participants to respond to the same questionnaire twice, the first time dishonestly according to specific instructions and the second time honestly. We gave the instructions in three specific contexts: (a) a child custody case, (b) a job interview for a sales manager position, and (c) a job interview for a position in a humanitarian organization. We used three faking contexts because faking changes according to the respondent’s objective. Having the same subject responding both dishonestly and honestly allows us to have a ground truth useful for evaluating the TF-IDF method’s accuracy level in identifying faked responses. Indeed, in most practical applications, only one set of responses per subject will be available, and the proposed approach’s goal is to spot faking patterns in the subject’s responses.

### Participants

The experimental procedure was approved by the ethics committee at the Department of General Psychology at the University of Padua (n. 4545). All participants in our experiments were native Italian speakers, and we administered the questionnaires online via social networks in August 2020 to a panel of subjects maintained by our department. We also collected information about the participants’ age, gender, and education level, which are shown in Table 1. The participants gave their written consent, which was digitally recorded along with the participants’ responses to the questionnaire.

**Table 1:** Number of participants and age and education of participants in the three groups.

Group	Number (Women)	Age	Education	Children
**CC**	221 (176)	41.7	15.4 (2.9)	Yes = 137/221
**JIS**	243 (185)	43.1	15.5(3)	–
**JIHO**	230 (188)	40.8	15.8 (2.8)	–

CC = child custody, JIS = job interview salesperson, JIHO = job interview humanitarian organization

### Procedure

In the three conditions, we asked participants to respond to the same set of 10 items twice, first altering their answers to obtain personal gain in the three contexts described above and then honestly. When a test is taken in a real faking-prone setting, the participant has not responded to the same test honestly before. The order faking first- honest second was adopted to have the faked condition close to the real administration condition.

All available responses were in multiple-choice format, and participants could choose only one option for each item. We asked the participants to respond by estimating their agreement using a 5-point Likert scale (where 1 indicates strong disagreement and 5 strong agreement) on the Italian version of the following 10 items from the Big Five Inventory [5, 13]. The English version of the test is reported below:

“I see myself as someone who …”

1. is outgoing, sociable (extroversion);
2. is reserved (extroversion reversed);
3. tends to find fault with others (agreeableness, reversed);
4. is generally trusting (agreeableness);
5. tends to be lazy (conscientiousness, reversed);
6. does a thorough job (conscientiousness);
7. gets nervous easily (emotional stability, reversed);
8. is relaxed, handles stress well (emotional stability);
9. has an active imagination (openness);
10. has few artistic interests (openness, reversed).

Before analyzing the collected data, we reversed the responses participants gave for reversed items (signaled above), so all the items’ final scores were directly comparable.

### Faking instructions

#### Group 1-CC

Pretend you and your spouse are separating and fighting in court for the custody of your child (imagine having a child if you don’t have one). A court-appointed psychologist will evaluate your parental skills and require you to respond to the following personality questionnaire. Answer the test in a way that emphasizes your parental skills, hiding behaviors or thoughts that are generally considered negative for a parent. Your goal is to get custody of your child.

#### Group 2-JIS

Pretend you are attending a job interview for the position of sales manager for a very prestigious company. It is your dream job, and you want to give the best possible image of yourself. Your goal is to obtain the job and to be considered better than the other candidates. Answer in a way that makes you look good, hiding behaviors or thoughts that are generally considered negative for the position you aspire to. Try to give a positive image of yourself, even if it means lying.

#### Group 3-JIHO

Participants received instructions similar to those we provided to Group 2, with the position being the only difference (a position in a humanitarian organization).

The procedure included a question to verify the participants read and fully comprehended the instructions in all three conditions before completing the questionnaire [14]. We did not include participants who did not answer this final question correctly in the analysis. The additional final question for Group 1 (Child custody litigation) was “What instructions have you received when filling in the questionnaire?” with the following response choices:

- I always had to tell the truth.
- I received no specific instructions.
- First I had to tell the truth, and then I had to lie to get custody of the children.
- I always had to lie to get custody of the children.
- First I had to lie to get custody of the children, and then I had to tell the truth.

Of the 258 participants, 37 did not satisfy this quality check. Therefore, we included only 221 participants in our analysis, discarding the responses of all participants who answered incorrectly.

The additional final question for Groups 2 and 3 was “What instructions have you received when filling in the questionnaire?” with the following response choices:

- I always had to tell the truth.
- I received no specific instructions.
- First I had to tell the truth, and then I had to lie to look good in a job interview.
- I always had to lie to make a good impression.
- First I had to lie to look good in a job interview, and then I had to tell the truth,

This quality check for Group 2 (JIS) resulted in the exclusion of 44 of 287 participants, with 243 participants included in the final analysis, who were selected based on their correct responses to the final questions. From Group 3 (JIHO), we excluded 42 of 272 participants, including 230 participants in the following evaluation.

The proposed approach to compute TF-IDF for questionnaire responses and the data employed for the evaluation are available in our public repository: https://github.com/albpurpura/TFIDF-Faking.

## Results and discussion

This section presents the results of the experiments we conducted to validate the proposed approach based on TF-IDF. We begin by giving a few characteristics of the data we collected and providing the analysis we performed with the raw data.

Table 2 shows the mean and standard deviation of the honest and dishonest responses in the three faking contexts. Data indicate that faking in different contexts resulted in changes to different items. For example, in the JIS dataset, faking did not significantly affect one of the two items related to a person’s agreeableness. In addition, we reported the probability of fakers to alter their response to each item in the different contexts we considered.

**Table 2:** Mean, SD and faking probability of the questions in each honest and dishonest questionnaire in different datasets.

Dataset	Measure	Q1	Q2	Q3	Q4	Q5	Q6	Q7	Q8	Q9	Q10
	Mean H	3.87	2.43	2.96	3.42	3.17	4.48	2.94	3.01	3.54	3.32
CC	Mean D	4.47*	2.20*	3.90*	3.69*	4.41*	4.58	4.35*	4.23*	3.40	3.98*
	SD H	0.90	0.96	0.99	0.97	1.22	0.68	1.22	1.09	1.10	1.25
	SD D	0.71*	0.93*	0.96*	0.90*	0.85*	0.81	0.80*	0.97*	1.24	0.93*
	Faking prob.	0.60	0.48	0.72	0.52	0.76	0.39	0.74	0.77	0.66	0.59
	Mean H	3.67	2.44	2.95	3.42	3.21	4.47	2.74	2.92	3.63	3.45
JIS	Mean D	4.55*	2.75*	3.46*	3.49	4.52*	4.67*	4.18*	4.40*	4.12*	3.84*
	SD H	0.97	1.09	1.04	1.06	1.20	0.77	1.18	1.14	1.04	1.26
	SD D	0.74*	1.25*	1.18*	1.04	0.82*	0.74*	1.05*	0.86*	1.06*	1.14*
	Faking prob.	0.67	0.61	0.77	0.62	0.74	0.39	0.79	0.80	0.70	0.62
	Mean H	3.77	2.38	3.00	3.55	3.21	4.49	2.81	3.00	3.56	3.42
JIHO	Mean D	4.53*	2.69*	3.94*	3.96*	4.47*	4.57	4.37*	4.35*	4.10*	4.08*
	SD H	0.95	1.04	1.11	0.96	1.25	0.71	1.16	1.18	1.10	1.26
	SD D	0.86*	1.25*	1.11*	0.94*	0.91*	0.86	0.84*	0.88*	1.02*	0.99*
	Faking prob.	0.64	0.63	0.71	0.65	0.73	0.38	0.77	0.76	0.66	0.54

Values in the faked questionnaire’s statistics marked with a * indicate a statistically significant difference (*p* < .05).

As Table 2 shows, different contexts affect different dishonest responses. For example, Q2 was changed in one direction in faking in the CC context and in the opposite direction in faking in a job interview. Moreover, the 10 items’ average scores did not differ significantly in the three honest-respondent groups. The faking probability per item shows that the likelihood of faking an item is strongly related to the faking context.

Although the probability of faking changes not only depends on the context but also on the item, we generally expected that the answers given in the faking conditions would score higher than those provided in the honest condition. In order to verify this, we calculated the proportion of responses that increased from the honest to the dishonest condition (e.g., from 3 to 5) or remained unchanged (Table 3). The overall pattern is similar in all three faking conditions. Most interestingly, in 13%–15% percent of the cases, dishonest responses were changed in an unexpected direction, with a lower score in the dishonest condition compared to the honest one.

**Table 3:** Unchanged and increased responses by group.

Group	Total responses	% Unchanged	% Increased
CC	2210	38%	48%
JIS	2430	33%	52%
JHO	2300	35%	52%

The table includes percentages of raw score responses that remained unchanged in the honest and dishonest conditions and changed in the expected direction according to the faking instructions. With the instructions we gave, we expected an increase in the raw scores (faking good).

This result could have occurred due to different faking strategies adopted by different participants and as well as test-retest issues. In fact, previous studies have shown that the test-retest reliability of the 10-item Big Five Questionnaire is 0.72 [5]. Since the reliability is 0.72, it could have led to the change in the answers between the dishonest and honest conditions. In other terms, the test-retest vagaries of non-faked responses could be at the origin of the decrease in response scores. Another possible explanation for the 15% decrease in faked responses may be related to the order of the presentation of the two versions that was Dishonest-Honest. Honest responses are given after each participant has already responded faking to the same test, which could have impacted the reliability of his/her honest responses.

**Fig 2:**
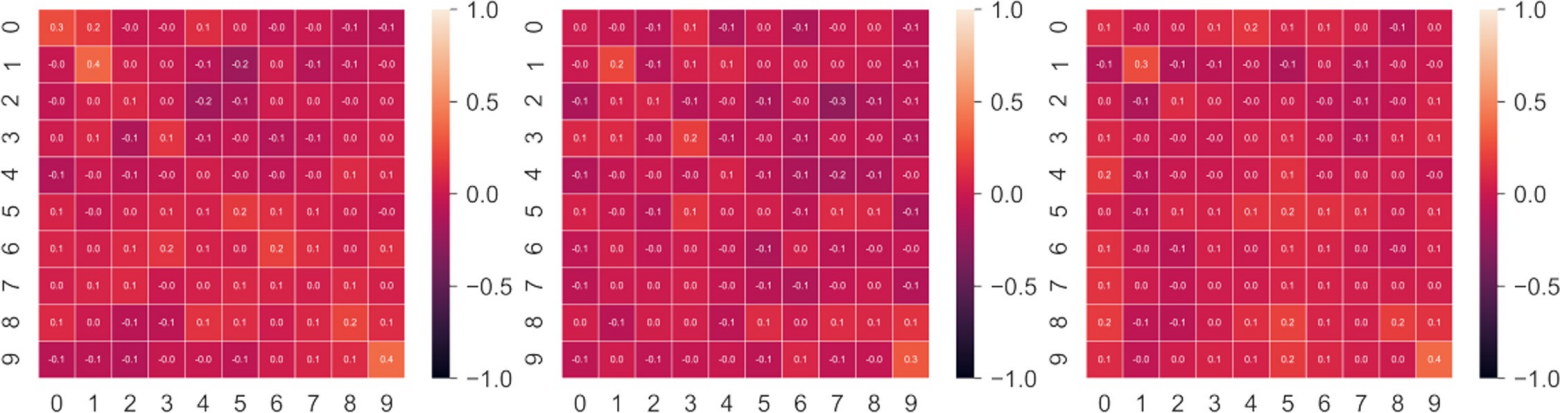
Correlation matrices. Correlation matrices between honest and dishonest responses in the CC, JIS and JIHO datasets respectively, from left to right. Correlation between responses in the honest and dishonest condition is virtually nonexistent, rendering the identification of the honest responses from the dishonest ones a difficult task.

In addition, we investigated the correlation between honest and dishonest answers. Fig 2 shows the correlation matrices between honest and dishonest responses in the three datasets. What emerges is that the correlations between honest and dishonest responses are around 0. This result indicates that, in all three groups, no linearity exists between honest and dishonest means; therefore, there is no straightforward procedure for predicting honest responses from dishonest ones.

### TF-IDF for item analysis

We changed the raw data into TF-IDF indices as described above to verify whether the TF-IDF approach can easily detect faked responses. As a first step, we verified whether the TF-IDF discriminates between honest and dishonest responses more efficiently than raw scores; as it can be seen in the correlation matrices, no linearity exists between the two distributions. For this purpose, we used the KL divergence, which is an entropy-based measure to compare probability distributions. The values of this measure range from 0 to infinity, where 0 indicates that the two considered distributions (e.g., honest and dishonest) completely overlap. Higher values of the KL-divergence indicate a higher degree of separation between honest- and dishonest-response distributions. The results reported in Tables 4–6 show much larger KL-divergence scores between the distributions of honest and dishonest responses for TF-IDF if compared with raw responses. This analysis highlighted that raw honest and dishonest distributions mostly overlap, while TF-IDF distributions show sensible differences with relatively higher KL-D values.

**Table 4:** KL divergence for CC dataset.

	Q1	Q2	Q3	Q4	Q5	Q6	Q7	Q8	Q9	Q10
Raw scores	0.37	0.04	0.54	0.07	1.00	0.08	1.05	0.96	0.03	0.27
TF-IDF	2.93	3.66	3.32	4.51	7.21	0.44	8.21	5.46	4.76	7.30

KL divergence between the response value distributions in the CC dataset of honest and dishonest responses for raw scores and TF-IDF. The closer to 0 the KL divergence is, the less distinguishable the two distributions are (honest and dishonest).

**Table 5:** KL divergence for JIS dataset.

	Q1	Q2	Q3	Q4	Q5	Q6	Q7	Q8	Q9	Q10
Raw scores	0.64	0.05	0.21	0.03	0.88	0.12	0.85	1.02	0.24	0.10
TF-IDF	1.29	2.23	1.08	4.38	2.86	0.44	2.65	3.38	2.95	3.28

KL divergence between the response value distributions in the JIS dataset of honest and dishonest responses for raw scores and TF-IDF. The closer to 0 the KL divergence is, the less distinguishable the two distributions are (honest and dishonest).

**Table 6:** KL divergence for JIHO dataset.

	Q1	Q2	Q3	Q4	Q5	Q6	Q7	Q8	Q9	Q10
Raw scores	0.58	0.06	0.44	0.11	0.71	0.13	1.09	0.84	0.16	0.23
TF-IDF	7.18	1.40	2.45	3.99	6.39	0.76	2.20	2.41	1.67	3.25

KL divergence between the response value distributions in the JIHO dataset of honest and dishonest responses for raw scores and TF-IDF. The closer to 0 the KL divergence is, the less distinguishable the two distributions are (honest and dishonest).

**Fig 3:**
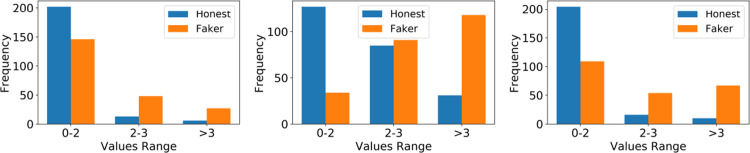
Average of TF-IDF response values over all 10 items for honest and dishonest response. Datasets from left to right: CC, JIS, JIHO. The odds for TF-IDF above 3 are the following: 1/3.8 (for every honest response with a TFIDF *>*3, there are 3.8 Fakers for CC), 1/6.7 (for JIS), and 1/4.5 (for JIHO). Transforming odds to probabilities yields the following probabilities of dishonesty for TF-IDF >3: 79.2% for CC, 87% for JIS, and 82% for JIHO.

Subsequently, to determine how the TF-IDF representation of honest responses differs from that of the dishonest ones, we calculated the average for each participant over all 10 items (see Fig 3). These results indicate that the average TF-IDF for fakers is significantly higher than for honest respondents. For example, in the JIS dataset, the distribution of the average TF-IDF is below 2, and we mainly observed higher values (>3) for dishonest responses. A TF-IDF above 3 indicates that responses are faked, with odds ranging from 3.8 to 6.7, depending on the context.

These results show that fakers can be distinguished from honest respondents based on the average of the TF-IDF over all the responses to the questionnaire items.

### Performance evaluation

TF-IDF acts as a novelty detector, with higher values indicating a response that a subject used frequently but all other participants used infrequently. The index is participant specific and item specific. However, TF-IDF can be used not only to spot fakers—as the results in Fig 3 indicate—but also to identify which items have been faked. As aforementioned, this feature is essential, as no procedure is available to address this problem. As we describe in more detail in the Material and Methods section, the proposed process for item-level faking detection can be summarized as follows:

1. Calculate the TF-IDF score for each response of a target participant.
2. Compare the obtained TF-IDF scores to a threshold specific for each questionnaire item.
3. If the TF-IDF values are outside a certain range, the response is categorized as an anomaly (faked).

**Fig 4:**
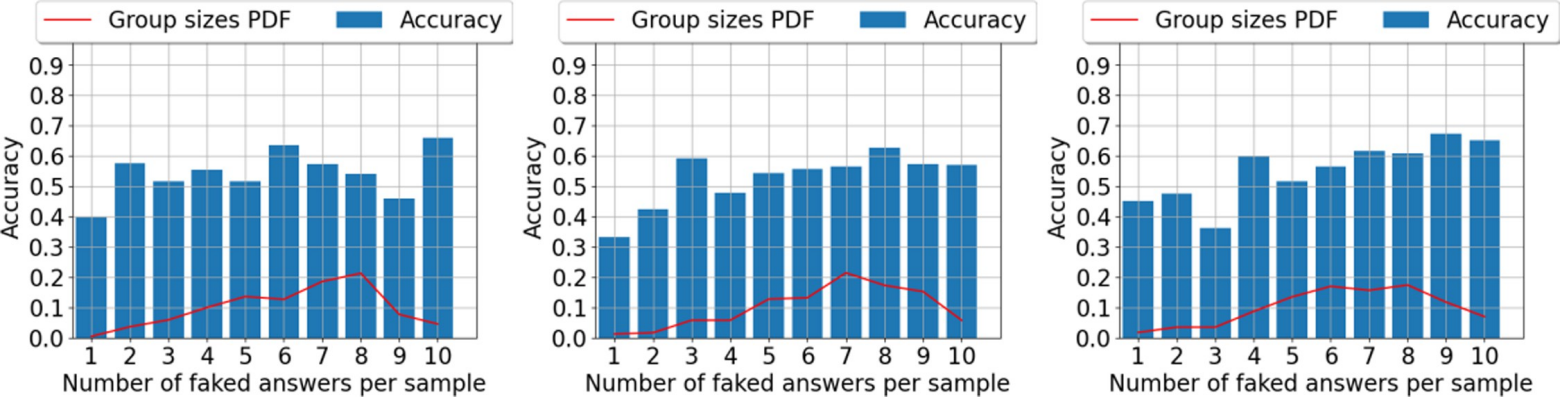
Probability density function. Probability density function (PDF) of the number of dishonest responses per questionnaire (red line) and per-item accuracy for the faking detection task using the TF-IDF model on various datasets: CC (left panel), JIS (central panel), and JIHO (right panel). The most frequent number of dishonest responses is 7/10. In each of the histogram’s bars, we report the average accuracy in identifying faked responses.

We applied this procedure to the three datasets (i.e., CC, JIS, JIHO), yielding the results summarized in Fig 4.

Given that each participant responded to the 10-item test twice, once in the dishonest condition and once in the honest one, we could identify the items with distorted responses for each participant. As indexed by the probability density function, fakers faked between 7 and 9 items more frequently, with few participants faking 1, 2, or 10 items (see Fig 3). We evaluated the proposed approach’s performance first in terms of accuracy (Fig 4), then in terms of precision, recall, and F1 Score (Table 7). We define the accuracy measure as the number of items on a questionnaire correctly identified as faked or nonfaked. Fig 4 shows the average accuracy we achieved with our TF-IDF approach in various scenarios.

**Table 7:** Performance evaluation.

	Group	Precision	Recall	F1 Score	Accuracy
TF-IDF	child custody, litigation (CC)	0.6700	0.4597	0.5046	0.5566
	job interview, sales manager (JIS)	0.7178	0.5279	0.5664	0.5642
	job interview, humanitarian organization (JIHO)	0.6808	0.5705	0.5877	0.5848
DM (raw scores)	child custody, litigation (CC)	0.4735	0.4176	0.4057	0.3516
	job interview, sales manager (JIS)	0.4692	0.3237	0.3413	0.3123
	job interview, humanitarian organization (JIHO)	0.4966	0.4295	0.4142	0.3422

Performance evaluation of the TF-IDF compared to the distribution-based model based on raw response values. The TF-IDF clearly outperforms the use of raw scores in all the evaluation metrics.

We observed that the accuracy remained relatively stable in the 0.4−0.6 range in all three conditions, indicating that faked items can be identified with the same level of accuracy independently of the specific faking strategy the subject used.

Table 7 shows the average performance in the three datasets. The results are compared to a simpler distribution-based model (DM), where dishonest responses are identified following the same procedure, but using raw scores. Results indicate a substantial increase in all the evaluative parameters for TF-IDF, compared to raw scores. Table 7 also shows the precision, recall, and F1 score metrics. They are defined as follows:

$$Precision=\frac{TP}{TP+FP}$$ (2)

$$Recall=\frac{TP}{TP+FN}$$ (3)

$$F1\;Score=\frac{2*Precision*Recall}{Precision+Recall}$$ (4)

where TP, FP, and FN indicate, respectively, the true positives (i.e., the number of dishonest responses correctly classified), false positives (i.e., the number of responses wrongly classified as dishonest), and false negatives (i.e., the number of dishonest responses our model did not catch), in our classification task [15].

**Fig 5:**
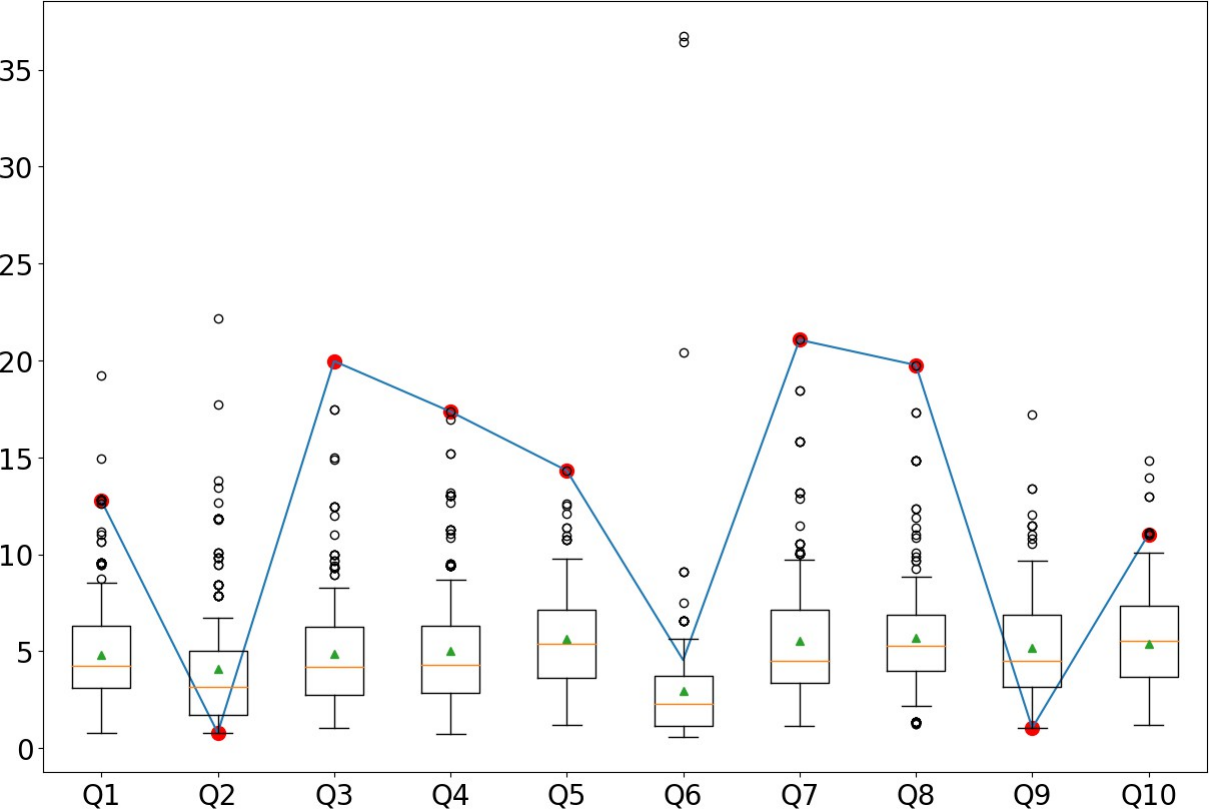
TF-IDF response values for participant 5 (dishonest responses) compared to the distributions of TF-IDF values of honest respondents in the CC dataset. The red dot indicates the items in which the participant faked. In this specific case, only Q6 received the same response in the honest and dishonest conditions. In this specific case, the algorithm accurately signaled seven of the nine dishonest responses. The total accuracy is 8/10. Two dishonest responses were missed.

**Fig 6:**
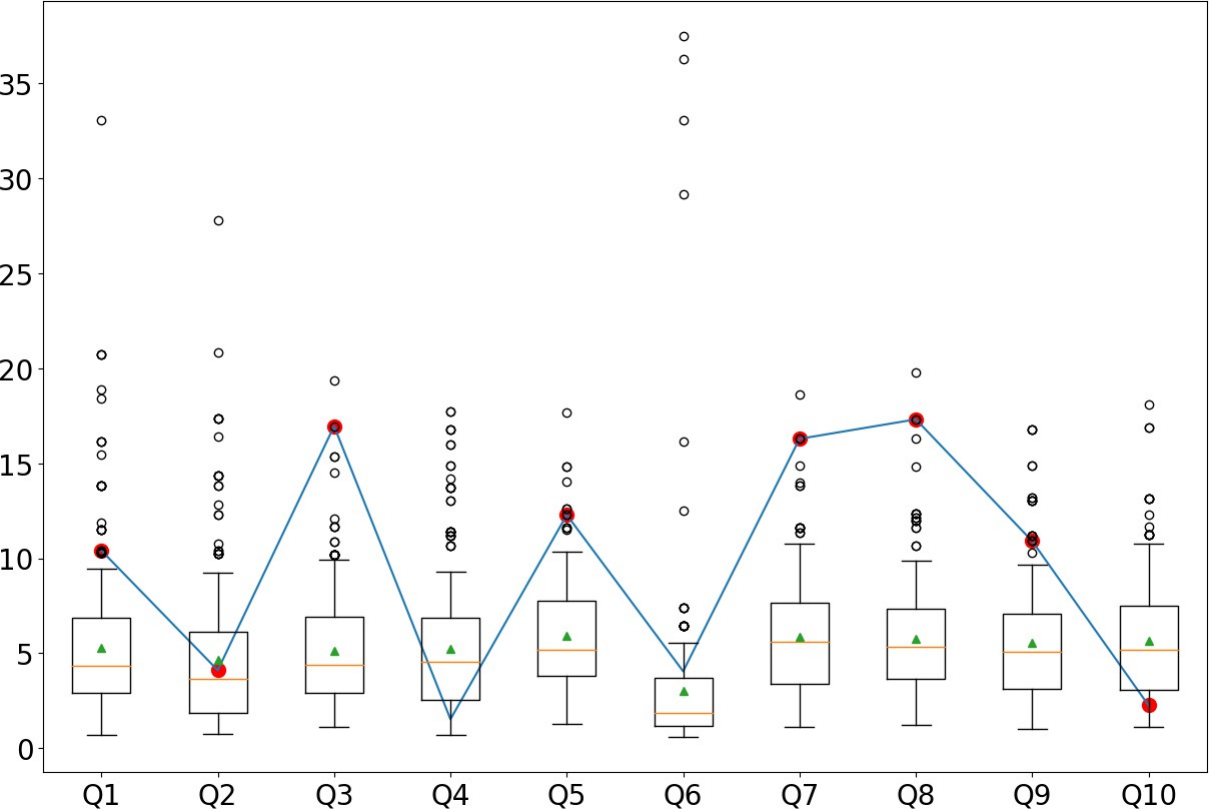
TF-IDF response values for participant 48 (dishonest responses) compared to the distributions of TF-IDF values of honest respondents in the JIS dataset. The red dots indicate the items in which the participant faked. In this specific case, Q4 and Q6 received the same response in the honest and dishonest conditions (i.e., the subject did not fake). Among the eight dishonest responses, the TF-IDF algorithm accurately spotted six of eight dishonest responses. The total accuracy is 8/10.

**Fig 7:**
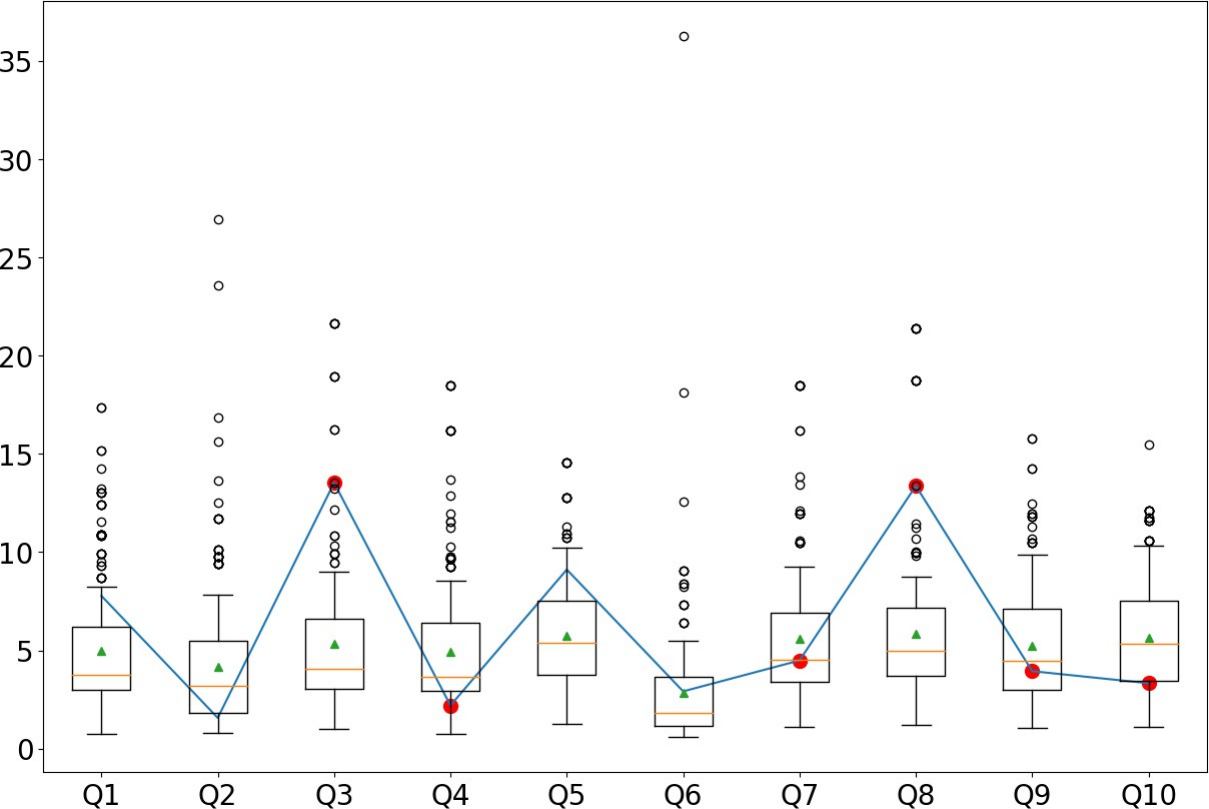
TF-IDF response values for participant 4 (dishonest responses) compared to the distributions of TF-IDF values of honest respondents in the JIHO dataset. Here we report a case where the precision is below the reported average. The red dots indicate the items in which the participant faked. In this specific case, Q1, Q2, Q5, and Q6 received the same response in the honest and dishonest conditions (i.e., the subject did not fake). Of the six dishonest responses, two were correctly identified. Q4, Q7, Q9, and Q10 were not reported as faked. The total accuracy is 6/10.

In practice, the TF-IDF can be used to analyze single-subject responses when no honest responses for a suspected faker are available. Figs 5–7 show the TF-IDF scores associated with the responses of three of our subjects (one for each dataset). In particular, we report the sequence of responses of three faking subjects (blue line) to each of the questionnaire’s 10 items. We compare each response to the distribution of honest responses participants gave for the same questionnaire item. The distribution of honest responses for each item is indicated in the figures with a box plot showing the mean of each distribution (orange line), the first quartiles (extremes of the boxes), and the 75th and 25th percentile values (upper and lower whiskers). Finally, we highlighted in red the responses where the participants changed their response when asked to fake. For example, for the participant reported in Fig 5 (Participant 5 from the CC group), only Q6 had the same response in the honest and dishonest conditions. In this specific case, the algorithm accurately detected seven of the nine dishonest responses when selecting the 75th percentile threshold for each item (i.e., when flagging as dishonest the responses that fall outside the 75th percentile of the distribution of honest responses to the same item). In this specific case, the total accuracy is 8/10 because the algorithm correctly identified all nonfaked responses (Q6) as honest but missed two faked ones (Q2 and Q9) that were incorrectly identified as honest.

Fig 6 illustrates the analysis of a single participant for the job interview for a sales manager position. In this case, the subject faked their responses in all but two items, Q4 and Q6, which were correctly identified as honest, and faked their responses in the remaining ones. Of the dishonest responses, the TF-IDF accurately detected six, incorrectly flagging two of them, Q2 and Q10, as honest, with an overall accuracy of 8/10.

Fig 7 shows a single example of a faker for landing a job in a humanitarian organization. Here, the subject faked their responses in six of 10 items, with the TF-IDF correctly identifying two of them, for an overall accuracy of 6/10 (i.e., it correctly identified the four honest responses and two of the dishonest ones, and it incorrectly classified four of the 10 responses in the questionnaire). Within this evaluation framework, we consider dishonest all responses that are different from the respective honest ones the same participant provided when taking the test honestly. However, as mentioned above, the test’s reliability is far from perfect [5]. Therefore, we believe that with a less strict identification of dishonest responses, the performance evaluation of our approach would yield higher values.

## Conclusions

Psychological tests usually require participants to endorse a statement describing a personality feature or behavior using a Likert scale (e.g., a 5-point scale). Respondents may easily fake responses on such tests by modifying their original, honest responses to achieve an objective (e.g., landing a job).

To handle this problem, faking propensity is usually detected using the so-called control scales, which are subsidiary scales added to personality scales (e.g., desirability scales, such as the Balanced Inventory of Desirable Responding (BIRD; [16]). However, these procedures help—at best—spot fakers but do not help specifically identify faked responses. In short, identifying specific items that have been faked (e.g., to obtain a job) is currently a major unsolved issue.

The task of identifying faking at the single-item level is challenging for the following reasons:

- the high variability of responses among individuals
- the various degrees of intensity of faking
- the objective of faking modulates where faking takes place and the degree of intensity (for example, faking to land a sales manager position requires faking in extroversion-related items, and faking on conscientiousness-related items may be a priority to land a bank position)

Currently, no technique is available to identify the specific faked responses. Control scales help identify only the participant’s general propensity to fake and do not pinpoint where faking actually takes place.

For these reasons, we propose the use of a new technique inspired by original research in information retrieval. The technique is known as term frequency-inverse document frequency [4] (TF-IDF) and is at the base of search engine technologies. When applied to search engines, this technique helps identify the most relevant web page with respect to a query [17]. This method has been used in cognitive neuropsychology to model semantic disorders [9].

In traditional item analysis, identical responses to the same item from two subjects are evaluated in the same way. In contrast, according to the TF-IDF modeling strategy, two identical responses (from two participants) to the same item may yield different evaluations, depending on other participants’ responses to the same items and the subject’s responses to the questionnaire’s other items. In other words, TF-IDF aggregates in a unique measure the distribution of the group responses to a target item as well as the subject’s response style (e.g., how frequently they also select a certain response value in other items).

TF-IDF, as presented here, can be considered a novelty detector. If the TF-IDF of a subject’s response to an item is abnormally high, it indicates that the participant’s response style does not belong to the sample of honest respondents. To verify the effectiveness of TF-IDF in identifying faked responses, we asked about 700 participants to respond to a short Italian version of the Big Five test (10-item Big Five questionnaire; [13]). We asked each participant to respond twice to the 10 items. The first time, we asked the participant to fake to achieve an advantage in one of three scenarios: (a) to obtain child custody, (b) to obtain a sales manager position, or (c) to obtain a position in a humanitarian organization. The second time, we asked the participant to respond honestly.

The exploratory analyses, performed on raw scores, indicated that

- faking (good) resulted in an increase in average response values on all but one of the 10 items and
- faking in different contexts, as expected, resulted in distortions of responses to different test items.

In evaluating the proposed TF-IDF approach, we observed the following:

- a TF-IDF based representation of item responses leads to a better separation between honest and dishonest responses than do raw scores;
- the average (of the 10 items of the considered questionnaire) TF-IDF response values of the honest respondents were lower than that of the dishonest ones;
- lower TF-IDF values characterize honest responses to single items, and larger values are characteristic of dishonest ones;
- the proportion of dishonest responses associated with large TF-IDF representations (e.g., above the 75th percentile of the honest-value distributions) was consistently larger than that of their honest counterparts;
- when applying the TF-IDF for item-level faking-detection, precision (the percentage of faked items correctly identified of all the faked items) was between 67% and 71%, indicating that TF-IDF correctly identifies most of the faked single-item responses. The same faking detection procedure, based on raw response values, instead yielded much lower precision values (see Table 7).

All the results indicate that TF-IDF can help catch deceptive responses at the single-item level with unprecedented accuracy. The identification of fakers and faking at the single-item level is achieved without the use of any control scale, only by capitalizing on one distinguishing feature of TF-IDF. It is worth noting that the use of TF-IDF as a novelty detector allows for the spotting of a faker if a group of honest respondents is available. This feature has interesting practical implications given that faking is context-specific and depends primarily on the faker’s strategy, which in turn depends on the objective that faking is expected to achieve (e.g., the custody of a child or landing a job position).

Finally, we have given examples of how the TF-IDF can be used to analyze a single participant’s responses (see Figs 5–7). When a suspected faker’s responses are analyzed, we do not have the ground truth of their honest responses. When the TF-IDF falls outside a given threshold (e.g., the 75th percentile of the TF-IDF of the honest responses), it may help determine whether a response is faked.

Although the present study was validated on the 10 items Big-five test questionnaire, its validity should be evaluated for other tests, as well. Since the role of the presentation order of the honest and dishonest condition has not been investigated here, future studies should balance the sample and provide further information on the possible priming effect.

Moreover, in this study, we asked participants to respond to three different scenarios and fake their test responses accordingly. The efficacy of the method should be confirmed also with high-stake test-takers in real settings rather than in low-stake instructed faking conditions as reported in this research.
